# First person – Christiaan Veerman

**DOI:** 10.1242/dmm.041202

**Published:** 2019-07-09

## Abstract

First Person is a series of interviews with the first authors of a selection of papers published in Disease Models & Mechanisms (DMM), helping early-career researchers promote themselves alongside their papers. Christiaan Veerman is first author on ‘Genetic variation in *GNB5* causes bradycardia by augmenting the cholinergic response via increased acetylcholine-activated potassium current (*I*_K,Ach_)’, published in DMM. Christiaan is a resident in cardiology in the lab of Prof. Bezzina at University of Amsterdam, The Netherlands, investigating pathophysiological mechanisms of inheritable cardiac arrhythmia syndromes using human induced pluripotent stem cells.


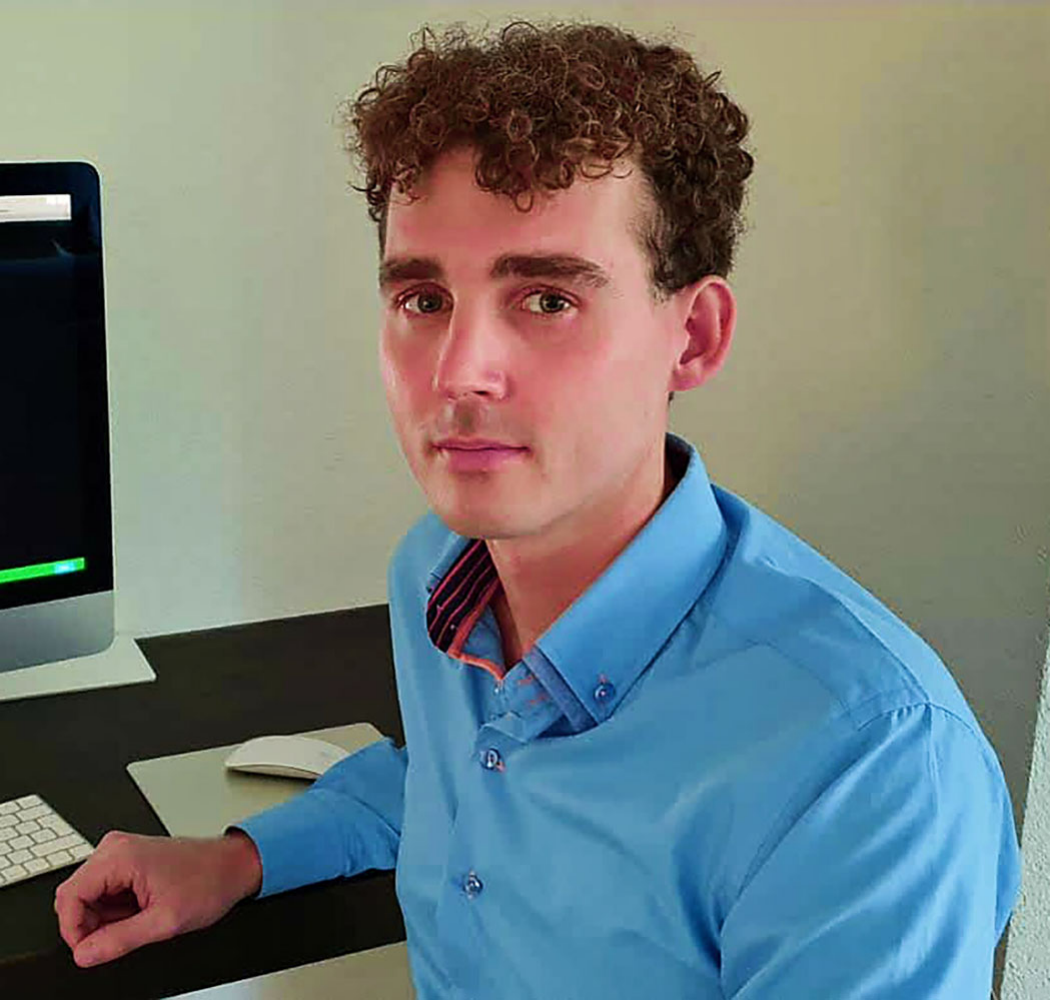


**Christiaan Veerman**

**How would you explain the main findings of your paper to non-scientific family and friends?**

In some families, genetic variations give rise to serious anomalies of the heart rhythm. Recently, a mutation in the gene *GNB5* was discovered that causes problems in the function of several organs, including a dangerously low heart rate (bradycardia). The mechanism by which this mutation causes bradycardia is unknown and the only therapy that is available is the implantation of a pacemaker. To increase our understanding and provide tools for therapy, we investigated the effects of the *GNB5* mutation. Ideally, one would study cardiomyocytes that are taken directly from patients; however, due to ethical and technical concerns this is not possible. An alternative, quite novel method is the use of human induced pluripotent stem cells (hiPSCs). hiPSCs are generated by reprogramming differentiated cells (e.g. dermal fibroblasts) into a pluripotent (undifferentiated) state, which enables them to differentiate into any cell type, including cardiomyocytes. By inserting the specific *GNB5* mutation in the genome of the (undifferentiated) hiPSCs, we could examine the effects of this mutation in cardiomyocytes. Because the gene product of *GNB5* is known to be involved in the function of a specific ion channel that regulates heart rate, we focused on the functional properties of this channel in the presence of either the mutant or wild-type *GNB5* gene product.

We found that the *GNB5* mutation results in an increased activity of this ion channel, which provides an explanation for the clinical features in patients. Moreover, cells that carried the mutation demonstrated a pronounced decrease in beating rate, which also reflects the clinical phenotype. Quite relevant was our finding that, by applying a drug that blocks the ion channel, we could reduce its activity and restore the decreased beating rate that was observed in mutant cardiomyocytes. The latter finding may have therapeutic implications: if these specific blockers also restore the normal heart rate in patients carrying the mutation, pacemaker implantation may not be necessary.

**What are the potential implications of these results for your field of research?**

Our study has provided insights into the molecular mechanism of bradycardia in patients carrying *GNB5* mutations. This is a very important step necessary for the development of therapies. In this regard we also have been able to identify a drug that, at the molecular and cellular level, provides a proof-of-principle for pharmaceutical therapy, which in the future may be helpful in patients that carry *GNB5* mutations.

“In our study, we used cardiomyocytes derived from hiPSCs. Importantly, these cells are human, providing a more faithful recapitulation of the *in vivo* human situation than other *in vitro* animal models.”

**What are the main advantages and drawbacks of the model system you have used as it relates to the disease you are investigating?**

In our study, we used cardiomyocytes derived from hiPSCs. Importantly, these cells are human, providing a more faithful recapitulation of the *in vivo* human situation than other *in vitro* animal models.

Generally, an important advantage of hiPSCs is the fact that they can be generated from patient material, ensuring that the genetic material in the cells is equal to the genetic material in the patient. In our study, patient cells were not available to us so we had to incorporate the *GNB5* mutation into the genome of a ‘wild-type’ hiPSC line using CRISPR/Cas9. The latter is a relatively new technology that allows the genome of hiPSCs to be modified in an efficient and precise way. Furthermore, the hiPSCs can be cultured and differentiated almost indefinitely, therefore providing enough cells to perform the necessary experiments.

**Figure:**
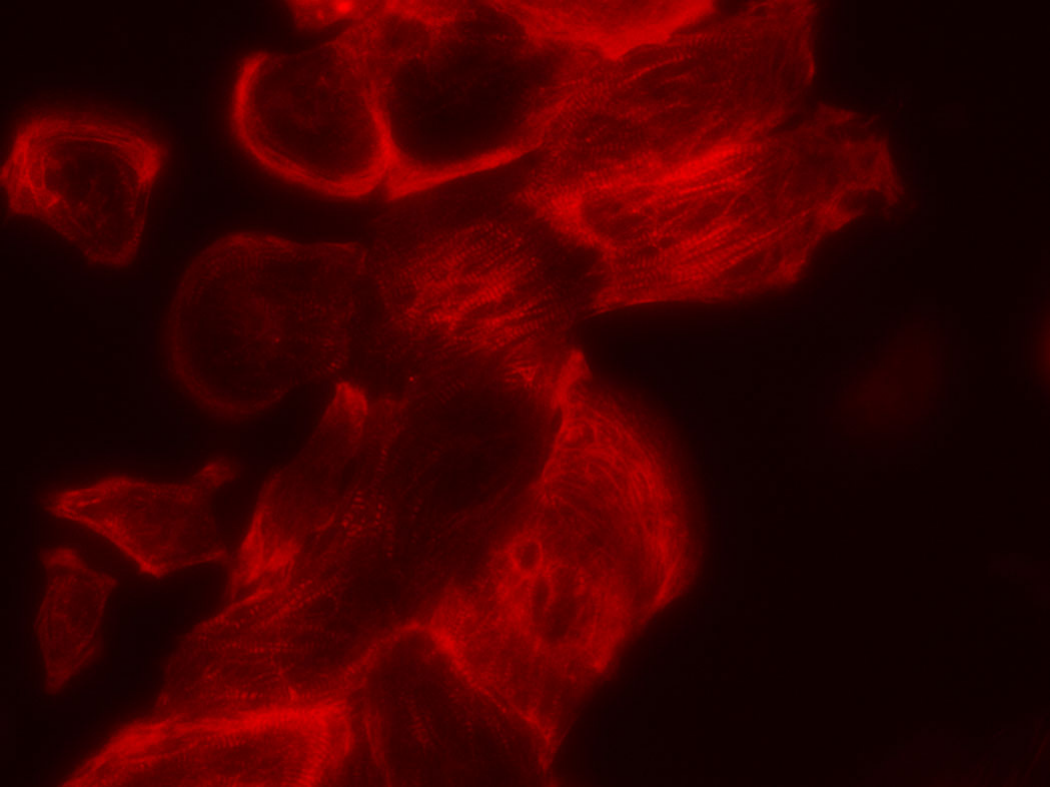
**Immunofluorescent staining of the sarcomeric protein troponine T in cardiomyocytes derived from human induced pluripotent stem cells (hiPSC-CMs), in which the specific genetic variant S81L in** ***GNB5*** **was implemented.**

Although the cardiomyocytes generated from hiPSCs (hiPSC-CMs) are human, some differences between these cells and cardiomyocytes from an adult person exist. These differences are mainly related to the fact that hiPSC-CMs are relatively immature and more similar to fetal cardiomyocytes. However, many new techniques are now being developed to improve hiPSC-CM maturation.

“The generation of heterogeneous tissues and organs from hiPSCs-derivatives is a field in active development.”

Furthermore, our study has focused on properties of single cells, while *in vivo* cardiomyocytes function in a tissue where important factors such as structure, non-cardiac cells, cellular alignment and heterogeneity of cardiomyocyte types play important roles. The generation of heterogeneous tissues and organs from hiPSCs-derivatives is a field in active development.

“Once again, the microscopic world of a single cell holds the key for understanding the functionality of a macroscopic pluricellular organism.”

**What has surprised you the most while conducting your research?**

Of course we are aware that a human being is a very complex pluricellular organism. Therefore, it was quite surprising and exciting to find out how much the behavior of a single cardiomyocyte derived from hiPSCs that were modified to introduce a specific mutation could reflect and in part explain the cardiac pathological characteristics of a patient carrying that specific mutation. Once again, the microscopic world of a single cell holds the key for understanding the functionality of a macroscopic pluricellular organism.

**Describe what you think is the most significant challenge impacting your research at this time and how will this be addressed over the next 10 years?**

An important drawback of the hiPSC-CM model is the fact that the generated cardiomyocytes are relatively immature. To date, different studies have focused on the development of techniques aimed at promoting the maturation of hiPSC-CMs (such as electrical stimulation, formation of 3D engineered heart tissue, metabolic maturation and maturation induced by chemically modified media). While some of these techniques have proven to be relatively successful, fully mature cardiomyocytes cannot be generated as yet. Continuous efforts probably involving the simultaneous combination of multiple strategies will eventually improve the phenotype of hiPSC-CMs to the point of more closely resembling the features of human, adult cardiomyocytes.

**What's next for you?**

Currently, I am training to become a cardiologist, which will take another 5 years. In the future, I have the ambition to combine clinical work with clinical and translational research.

## References

[DMM041202C1] Veerman C. C., Mengarelli I., Koopman C. D., Wilders R., van Amersfoorth S. C., Bakker D., Wolswinkel R., Hababa M., de Boer T. P., Guan K. et al. (2019). Genetic variation in *GNB5* causes bradycardia by augmenting the cholinergic response via increased acetylcholine-activated potassium current (*I*_K,Ach_). *Dis. Model. Mech.* 12, dmm037994 10.1242/dmm.037994 31208990 PMC6679373

